# ATGL deficiency aggravates pressure overload-triggered myocardial hypertrophic remodeling associated with the proteasome-PTEN-mTOR-autophagy pathway

**DOI:** 10.1007/s10565-022-09699-0

**Published:** 2022-02-26

**Authors:** Xiao Han, Yun-Long Zhang, Qiu-Yue Lin, Hui-Hua Li, Shu-Bin Guo

**Affiliations:** 1 Department of Emergency Medicine, Beijing Key Laboratory of Cardiopulmonary Cerebral Resuscitation, Beijing Chaoyang Hospital, Capital Medical University, Beijing, 100020 China; 2 Department of Cardiology, Institute of Cardiovascular Diseases, First Affiliated Hospital of Dalian Medical University, Dalian, 116011 China

**Keywords:** ATGL, Cardiac remodeling, Proteasome, PTEN, mTOR, Autophagy

## Abstract

Persistent myocardial hypertrophy frequently leads to heart failure (HF). Intramyocardial triacylglycerol (TAG) accumulation is closely related with cardiac remodeling and abnormal contractile function. Adipose triglyceride lipase (ATGL), a key enzyme in TAG metabolism, regulates cardiac function. However, its associated molecular pathways have not been fully defined. Here, cardiac hypertrophy and HF were induced in wild-type (WT) or ATGL knockout (KO) mice through transverse aortic constriction (TAC) for up to 4 weeks. TAC in WT mice significantly reduced cardiac function and autophagy while enhancing left ventricular hypertrophy, interstitial fibrosis, inflammatory response, superoxide generation, and cardiomyocyte apoptosis, accompanied with upregulation of the proteasome activity, reduction of PTEN level and activation of AKT-mTOR signaling, and these effects were further aggravated in ATGL KO mice. Interestingly, ATGL KO-mediated cardiac dysfunction and remodeling were markedly reversed by proteasome inhibitor (epoxomicin) or autophagic activator (rapamycin), but accelerated by PTEN inhibitor (VO-OHpic) or autophagy inhibitor 3-MA. Mechanistically, ATGL KO upregulated proteasome expression and activity, which in turn mediates PTEN degradation leading to activation of AKT-mTOR signaling and inhibition of autophagy, thereby enhancing hypertrophic remodeling and HF. In conclusion, ATGL KO contributes to TAC-induced cardiac dysfunction and adverse remodeling probably associated with the proteasome-PTEN-mTOR-autophagy pathway. Therefore, modulation of this pathway may have a therapeutic effect potential for hypertrophic heart disease.

**Graphical abstract:**

TAC-induced downregulation of ATGL results in increased proteasome (β1i/β2i/β5i) activity, which in turn promotes degradation of PTEN and activation of AKT-mTOR signaling and then inhibits autophagy and ATP production, thereby leading to cardiac hypertrophic remodeling and dysfunction. Conversely, blocking proteasome activity or activating autophagy attenuates these effects.

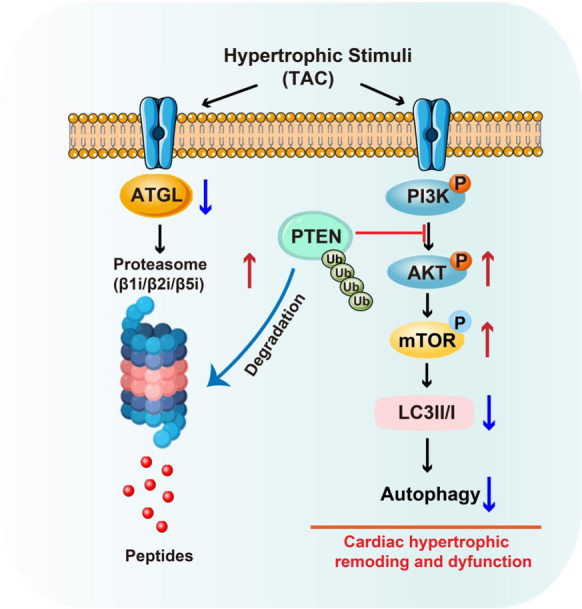

**Supplementary Information:**

The online version contains supplementary material available at 10.1007/s10565-022-09699-0.

## Introduction

Sustained pressure overload frequently triggers myocardial hypertrophy and eventually resulting in heart failure (HF). Cardiac hypertrophy presents with activation of fetal gene expression, protein synthesis, increased cell size, and actin reorganization (Heineke and Molkentin 2006). Several signaling pathways, including PTEN/AKT/mTOR, MAPK, and calcineurin/NFAT signaling pathways regulate cardiac hypertrophy (Heineke and Molkentin 2006). PTEN, a negative regulator of the AKT-mTOR signaling cascade, is essential in maintaining cardiac homeostasis, and its loss promotes pressure overload-induced cardiac pathological remodeling and contractile defects (Roe et al. 2015; Shi et al. 2020; Xu et al. 2014). Moreover, mTOR is a critical regulator of autophagy and cardiac hypertrophic signaling (Kim et al. 2018; Li et al. 2016; Shi et al. 2020; Xu et al. 2014). Therefore, modulation of PTEN/mTOR signaling may have therapeutic effects against cardiac hypertrophy and dysfunction.

Myocardial triacylglycerol (TAG), which is stored in cytosolic lipid droplets, is a highly dynamic fatty acid storage pool and energy source of the heart. In patients with hypertrophic heart disease, myocardial TG level is negatively associated with cardiac left ventricle (LV) mass and stroke volume in patients with cardiac hypertrophy (Sai et al. 2017). In multiple tissues, including the heart, ATGL (adipose triglyceride lipase or PNPLA2) regulates TAG lipolysis by hydrolyzing fatty acids from TAG stores (Kintscher et al. 2020). ATGL is highly expressed in various tissues, including brown and white adipose tissues, muscles, and heart tissues (Kintscher et al. 2020). Global knockout of the ATGL gene induces severe lipotrophic cardiomyopathy, leading to premature mortality (Haemmerle et al. 2006). Human ATGL mutations cause myocardial steatosis and dysfunction (Hirano et al. 2008). In mice models of cardiac hypertrophy and in in vitro phenylephrine-treated cardiomyocytes, ATGL levels were found to be suppressed (Gao et al. 2015), which regulated cardiac dysfunction and remodeling by affecting lipid and energy metabolism. ATGL knockout (KO) enhanced ceramide and intracellular free fatty acid accumulation, inducing by pressure overload stress (Kienesberger et al. 2012). Moreover, ATGL deficiency in mice suppressed PPAR-α and PGC-1 levels and severely impaired mitochondrial substrate oxidation, resulting in lipid deposition and cardiac dysfunction (Haemmerle et al. 2011). Myocardial ATGL KO alters TAG and energy metabolism, causing cardiac dysfunction. In contrast, overexpression of cardiac-specific ATGL markedly reduced myocardial TAG levels and the rate of fatty acid oxidation, preventing the occurrence of pressure overload-induced heart remodeling and systolic dysfunction (Kienesberger et al. 2012). However, it has not been established whether other molecular mechanisms regulate the effects of ATGL on pressure overload-induced HF.

In this study, we showed that ATGL KO significantly enhances TAC-induced cardiac remodeling and HF associated with suppression of autophagy via the proteasome-PTEN-mTOR signaling. These findings uncover a new mechanism through which ATGL regulates cardiac function, implying that ATGL may represent a novel target for the treatment of hypertrophic disease.

## Material and methods

### Animals and treatment

C57BL/6 wild-type (WT) and global ATGL knockout (KO) mice (019,003-B6;129P2-Pnpla2tm1Rze/J) were obtained from Jackson laboratory (Bar Harbor, ME, USA) and SPF Biotechnology Co., Ltd. (Beijing, China). In the ATGL KO strain, exon 1 of the Pnpla2 gene was replaced with a NEO cassette. The heterozygotes were viable and fertile, while the homozygotes exhibited a shortened lifespan (14–16 weeks) and died from severe cardiac steatosis and lethal myopathy (Haemmerle et al. 2006). Moreover, homozygotes exhibit impaired lipid homeostasis, glucose tolerance, and energy homeostasis (Trites and Clugston 2019). All animals were housed in a conditioned room with free access to receive sterilized standard mouse diet and drank water freely.

### Establishment of cardiac remodeling mice models and inhibitor administration

Cardiac hypertrophic mice models were established via transverse aortic constriction (TAC), as previously reported (Chen et al. 2019; Lin et al. 2021; Xie et al. 2019). Briefly, ATGL KO and WT mice (male, 8 weeks old, *n* = 8 per group) were anesthetized by 2.0% isoflurane injection. Then, the left chest wall of the mice was opened the transverse aorta ligated (7.0 nylon suture), resulting in 65–70% constriction after needle removal. Mice in the sham control group were received with the same procedures, but without ligation.

To activate or inhibit autophagy, animals (*n* = 8 per group) were intraperitoneally injected with the mTOR inhibitor, rapamycin at 4 mg/kg, or with 3-MA at 10 mg/kg daily for 4 weeks (Yang et al. 2020, 2021). For proteasome or PTEN inhibition, mice (*n* = 6 per group) were intraperitoneally injected with epoxomicin at 2.9 mg/kg or VO-OHpic at 10 mg/kg daily for 4 weeks (Han et al. 2020; Meng et al. 1999). These agents had been obtained from Selleck (Houston, TX, USA) and dissolved in 0.9% saline. Control mice were administered with similar volumes of saline. After treatment for 4 weeks, animals were anesthetized with 100 mg/kg sodium pentobarbital (Sigma-Aldrich, Dorset, UK), after which they were sacrificed, and their hearts obtained collected for further analyses.

### Echocardiographic assessment

Four weeks after TAC, transthoracic echocardiographic measurements of mice were performed using a 30-MHz ultrasound system (Vevo 1100, VisualSonics, Toronto, ON, Canada). Several cardiac parameters were recorded as previously reported (Xie et al. 2019). The pulsed-wave Doppler was used for simultaneous recording of aortic arch blood velocities (Deng et al. 2021). Transaortic pressure gradients were analyzed by pulsed-wave doppler examination using the equation: Pressure gradient is equal to 4 × velocity2. LV hemodynamics were determined using 1.4-F Millar pressure–volume catheters (SPR-839) (Chen et al. 2019).

### Histopathological examination

Mice were anesthetized using 100 mg/kg sodium pentobarbital (Sigma-Aldrich, Dorset, UK), heart tissues resected, fixed in 4% paraformaldehyde (PFA) for 24 h, paraffin-embedded and then sliced to 5 μm-thick secrions. Staining of H&E (haematoxylin–eosin) or Masson trichrome was performed on tissue slices (Chen et al. 2019; Xie et al. 2019; Zou et al. 2019). Cross-sectional areas of LV myocytes were evaluated by staining with rhodamine-labeled WGA (wheat germ agglutinin, 50 μg/mL) for 60 min (Chen et al. 2019; Xie et al. 2019; Zou et al. 2019). Images were obtained from cardiac samples (> 20 random fields per sample) at × 100 magnification. Cardiac myocyte areas were determined by analysis of 200 cells per sample. Immunohistochemistry (IHC) was performed using antibodies against α-smooth muscle actin (α-SMA) (Abcam, Cambridge, MA, USA) or Mac-2 (Santa Cruz, Dallas, Texas, USA). The oil red O staining assay was performed to assess lipid deposition on 10 μm-thick heart sections(Liao et al. 2020). Image Pro Plus 3.0 software was used for imaging analysis.

### TUNEL staining for cardiomyocyte apoptosis

Cell apoptosis was detected by using the in situ cell death detection assay (Roche, Basel, Switzerland) according to protocols as described (Xie et al. 2019). The TUNEL kit was used to stain heart sections. Cardiomyocytes and nuclei were counterstained with antibodies against α-actinin and DAPI (Sigma-Aldrich, Dorset, UK), respectively. Then, heart sections were imaged in 6–8 random fields of view with proportions of TUNEL-positive nuclei calculated as previously described (Xie et al. 2019).

### Tissue and serum lipid analysis

Myocardial tissues were homogenized in a cold lysis buffer containing a protease inhibitors. Homogenates were cleared with centrifugation (1,200 × *g*) for 20 min at 4 °C. Protein levels were measured with the BCA assay kit. Heart TG levels were evaluated using the TG assay kit (Solarbio, Beijing, China) while common serum lipoproteins were measured using the resuspended lipid extracts in ice-cold 1% Triton X-100 via the colorimetric assay kit (Elabscience, Wuhan, China).

### Quantitative real-time PCR analysis

Tissue RNAs were purified from cardiac left ventricles using the TRIzol buffer (Invitrogen, Carlsbad, CA, USA). Briefly, 1–2 μg cDNA was used for first-strand cDNA synthesis. qPCR was performed on a Bio-Rad iCycler IQ system (Chen et al. 2019; Xie et al. 2019; Zou et al. 2019) using gene-specific primers for interleukin (IL)-6, IL-1β, BNP, ANF, Collagen I, and III. GAPDH was the reference gene. The primers used for qPCR analysis are illustrated in Supplemental Table 1.

### Immunoblotting analysis

Total protein extracts were obtained from frozen heart tissues using the RIPA lysis buffer and quantified with the BCA assay method (Thermo Fisher, Carlsbad, CA, USA). Approximately 40–60 μg proteins were resolved on 8–10% SDS-PAGE and transferred onto polyvinylidene fluoride membranes that were then blotted overnight with appropriate primary antibodies at 4 °C for 12 h (Chen et al. 2019; Xie et al. 2019; Zou et al. 2019). Then, membranes were developed on a Gel-pro Analyzer 4.5 (Media Cybernetics). The antibodies used in this study are shown in Supplemental Table 2.

### Culture of primary cardiomyocytes and the autophagic flux assay

For the isolation of neonatal rat cardiac myocytes (NRCMs), hearts obtained from 1–2-day-old SD rats were sliced into > 12 pieces and dissociated with trypsin (0.25%). The isolated cardiomyocytes were cultured in DMEM/F12 plus with 10% FBS for 24 h. Then, the FBS-containing medium was changed with DMEM/F12 without FBS (Xie et al. 2019).

The autophagic flux assay was performed to evaluate mRFP-LC3 and GFP-LC3 puncta localization. GFP and RFP tags were fused to the C-termini of the autophagosome marker, LC3 (mRFP-GFP-LC3). This assay allows the degradation of acid-sensitive GFP in autolysosomes while the stability of the acid-insensitive RFP is maintained. The yellow puncta indicate autophagosome, while the red puncta represent autolysosomes. The autophagic flux was given by the ratio of autophagosomes (yellow puncta) to autolysosomes (LC3B red puncta) (Xie et al. 2019).

First, NRCMs were infected with the adenovirus expressing mRFP-GFP-LC3 along with siRNA-ATGL (Ad-siRNA-ATGL) without GFP, or the scramble siRNA-control (MOI = 50) for 24 h. Then, they were pretreated with rapamycin (RAPA, 20 nM, mTOR inhibitor), 3-MA (5 mM, autophagy inhibitor), VO-OHpic (50 nM, PTEN inhibitor), or epoxomicin (100 nM, proteasome inhibitor) and then stimulated with 100 nM Ang II for 48 h (Bu et al. 2020; Chen et al. 2019; Meng et al. 1999; Yan et al. 2019). LC3-positive puncta were examined by confocal microscopy (Leica STELLARIS 5 Microsystems, Wetzlar, Germany). Then, counts of autophagosomes (yellow) and autolysosomes (red) in each condition (20–30 cells per group) were analyzed with the ImageJ (NIH, Bethesda, MD, USA).

### Evaluation of the proteasome activities

The fluorogenic peptide substrates, including Z-LLE-AMC (45 μmol/L), Ac-RLRAMC (40 μmol/L), and Suc-LLVY-AMC (18 μmol/L) were used to measure cardiac proteasome caspase-like, trypsin-like and chymotrypsin-like activities, respectively (Chen et al. 2019; Xie et al. 2019; Zou et al. 2019). Briefly, 20 μg of tissue proteins was added to 100 μL of 50 mM HEPES (pH 7.5, 5 mmol/L MgCl2, 20 mmol/L KCl, and 1 mmol/L DTT), after which they were incubated with fluorogenic peptide substrates in the presence or absence of the proteasome inhibitors epoxomicin (5 μmol/L) or MG132 (20 μmol/L) at 37℃ for 10 min. Fluorescence intensities were assessed at the excitation and emission wavelengths of 380 nm and 460 nm.

### Statistical analysis

All results are shown as the mean ± SEM. Comparisons of between groups means were performed with the unpaired two-tailed Student’s *t* test, while comparisons of means among groups were conducted with one-way ANOVA followed by Newman-Keuls for multiple comparisons. GraphPad Prism 5.0. was set as the threshold for statistical significance.

## Results

### ATGL KO aggravates TAC surgery-induced myocardial dysfunction

**Fig. 1:**
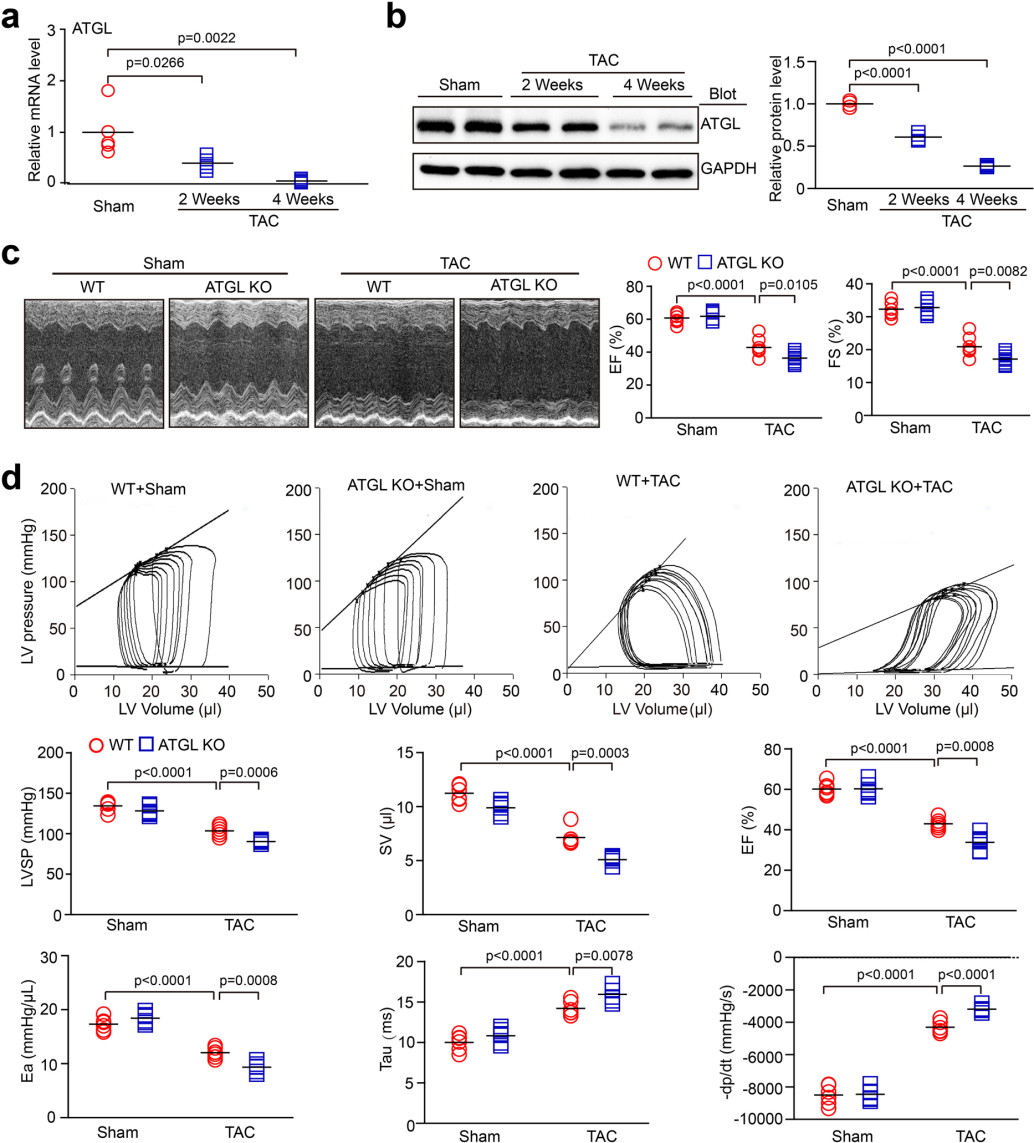
Ablation of ATGL aggravates pressure overload-induced cardiac dysfunction. Wild-type (WT) and ATGL knockout (KO) mice were subjected to sham or transverse aortic constriction (TAC) operation for 2–4 weeks. **a**, qPCR analysis of the ATGL mRNA level in WT hearts at weeks 2 and 4 of TAC operation (*n* = 5). **b**, Immunoblotting analysis of the ATGL protein level in WT heart (*n* = 4). **c**, Representative echocardiographic measurement of left ventricular (LV) chamber (left), and the calculation of LV ejection fraction (EF%) and fractional shortening (FS%) after 4 weeks of TAC operation (right, *n* = 8). **d**, Pressure–volume analysis of systolic and diastolic function. Representative LV pressure–volume loops in each group (top). Summary data on systolic function and diastolic function (bottom, *n* = 6). LVSP, LV systolic pressure; SV, stroke volume; EF, ejection fraction; Ea, arterial elastance; Tau, relaxation time constant; -dP/dt, maximal rate of pressure decline (diastolic indexes). Data are presented as mean ± SEM, and n represents number of animals

To assess the role of ATGL in the regulation of myocardial hypertrophic remodeling and function, WT and ATGL KO mice were subjected to TAC surgery for 2 to 4 weeks to establish in vivo model of pressure overload-induced hypertrophic remodeling and dysfunction. At 1 week post TAC surgery, hemodynamic measurements revealed that the transaortic pressure gradient was significantly elevated in TAC-operated ATGL KO or WT mice, compared to sham group mice, but was comparable between 2 groups following sham or TAC surgery (Supplementary Fig. 1a). Meanwhile, compared to baseline control, ejection fraction percentage (EF%) and fractional shortening percentage (FS%), which are indices for cardiac contractile function, were markedly elevated in WT mice until week 2, and then decreased at weeks 3–4 following TAC surgery. However, in ATGL KO mice, cardiac dysfunction time-dependently decreased and peaked at week 4 (Supplementary Fig. 1b). Moreover, ATGL mRNA and protein levels in TAC-operated hearts were time-dependently suppressed compared to sham-operated controls (Fig. 1a–b). Echocardiography revealed that relative to the sham group, TAC-treated WT mice exhibited markedly reduced of cardiac contractile functions (decreased FS% and EF%), and that these parameters were further reduced in ATGL KO mice (Fig. 1c). Then, we assessed the effects of ATGL KO on cardiac functions using invasive pressure–volume analysis. After 4 weeks of TAC surgery, there was no sudden death or cardiac arrhythmia in ATGL KO or WT mice. However, TAC-operated WT mice displayed a rightward shift of end-systolic pressure–volume (P–V) relation accompanied by reduced left ventricle systolic pressure (LVSP), stroke volume (SV), EF, as well as arterial elastance (Ea), and increased Tau and maximal rate of pressure decrease (− dP/dt). These effects were more pronounced in ATGL KO mice (Fig. 1d; Supplemental Table 3), but were not significantly different between ATGL KO and WT animals after sham operation (Fig. 1c–d). These findings imply that ATGL KO promotes pressure overload-induced cardiac dysfunction.

### ATGL deficiency aggravated TAC-induced cardiac inflammation, fibrosis, and hypertrophy

**Fig. 2:**
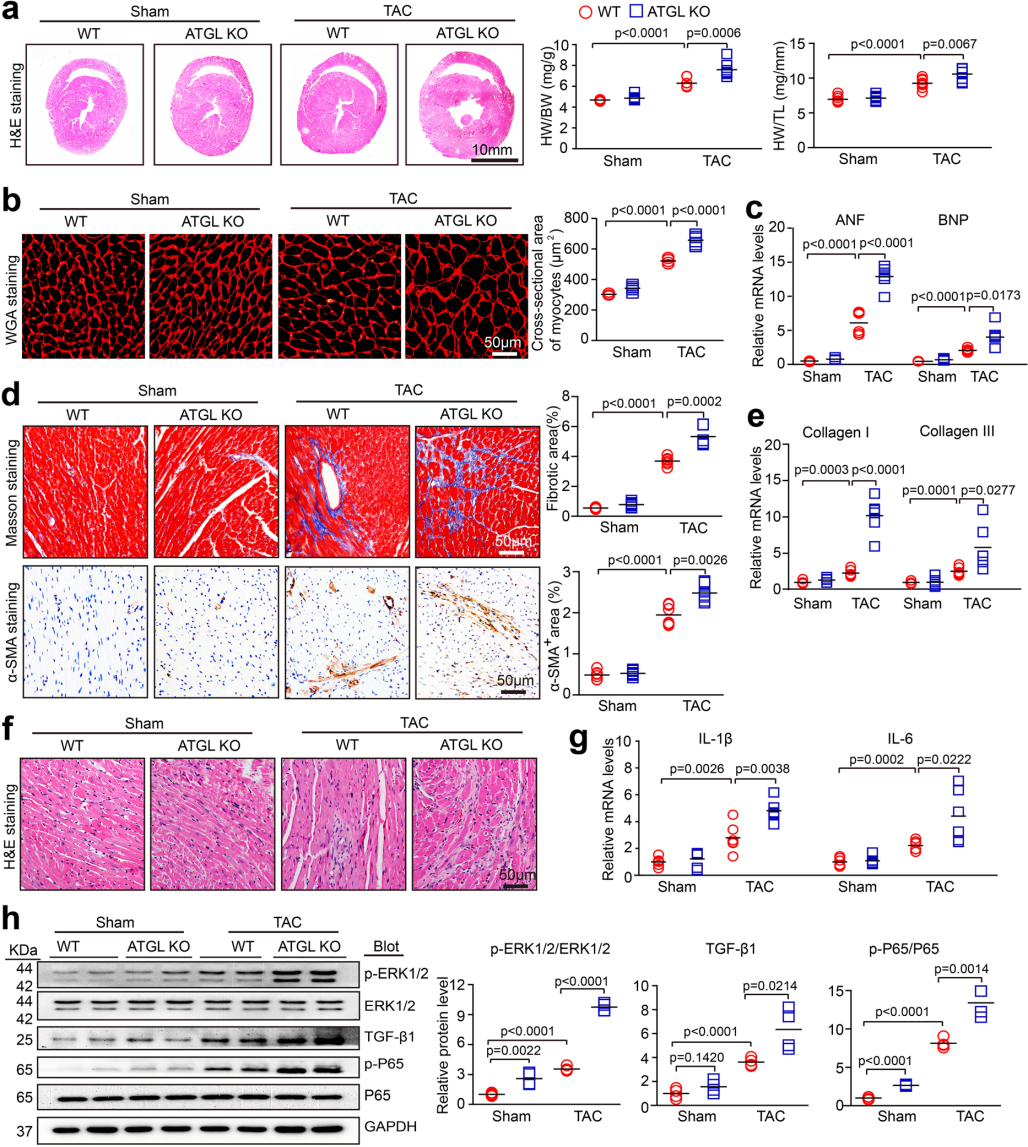
Knockout of ATGL promotes pressure overload-induced cardiac hypertrophic remodeling. **a**, WT and ATGL KO mice were subjected to sham or TAC operation for 4 weeks. Representative images of heart sections (left). Scale bar: 10 mm. The ratios of heart weight to body weight (HW/BW) and heart weight tibial length (HW/TL) (right, *n* = 8). **b**, Representative images of TRITC-labeled wheat germ agglutinin (WGA) staining to measure cardiac myocyte size (left). Quantification of cross-sectional area of myocytes (right, *n* = 6, 150–200 cells counted per sample). **c**, qPCR analyses of ANF and BNP mRNA levels in the heart (*n* = 6). **d**, Representative images of Masson’s trichrome and immunohistochemical staining to examine fibrosis (top) and the number of myofibroblasts (bottom), respectively. Quantification of fibrotic area (%) and the percentage of α-SMA-positive myofibroblasts (%) (right, *n* = 6). **e**, qPCR analyses of Collagen I and Collagen III mRNA levels (*n* = 6). **f**, Representative images of H&E staining of heart sections. Scale bar: 50 μm. **g**, qPCR analyses of IL-1β and IL-6 mRNA levels (*n* = 6). **h**, Immunoblotting analyses of p-ERK1/2, ERK1/2, TGF-β1, p-P65, P65, and GAPDH in the heart (left). Quantification of the relative protein levels (right, *n* = 4). Data are presented as mean ± SEM, and n represents number of animals

Next, we determined if ATGL KO affects cardiac remodeling. Relative to sham group, TAC for 4 weeks in WT mice markedly accelerated myocardial hypertrophy, as shown by higher ratios of heart weight/body weight (HW/BW) and heart weight/tibia length (HW/TL), left ventricular (LV) wall thickness, cardiac myocyte size, and the mRNA levels of ANF and BNP. These effects were more enhanced in TAC-treated ATGL KO mice (Fig. 2a–c). Furthermore, TAC-induced elevations in LV fibrosis and inflammatory responses were observed in WT mice, as indicated by increased fibrotic areas, staining density of α-SMA, inflammatory cell infiltrations as well as the mRNA expressions of proinflammatory cytokines (IL-1β and IL-6) and fibrotic markers (Collagen I and III) in TAC-operated ATGL KO hearts, relative to TAC-operated WT hearts (Fig. 2d–g). In addition, TAC surgery highly upregulated ERK1/2 and P65 phosphorylation levels and TGF-β1 protein level in WT hearts, and this upregulation was even higher in TAC-operated ATGL KO hearts (Fig. 2h). There were similar changes in these parameters for myocardial hypertrophy, fibrosis, inflammation between two groups following sham surgery (Fig. 2a–h).

### ATGL KO accelerated TAC-induced oxidative stress and cardiomyocyte apoptosis in mice

**Fig. 3:**
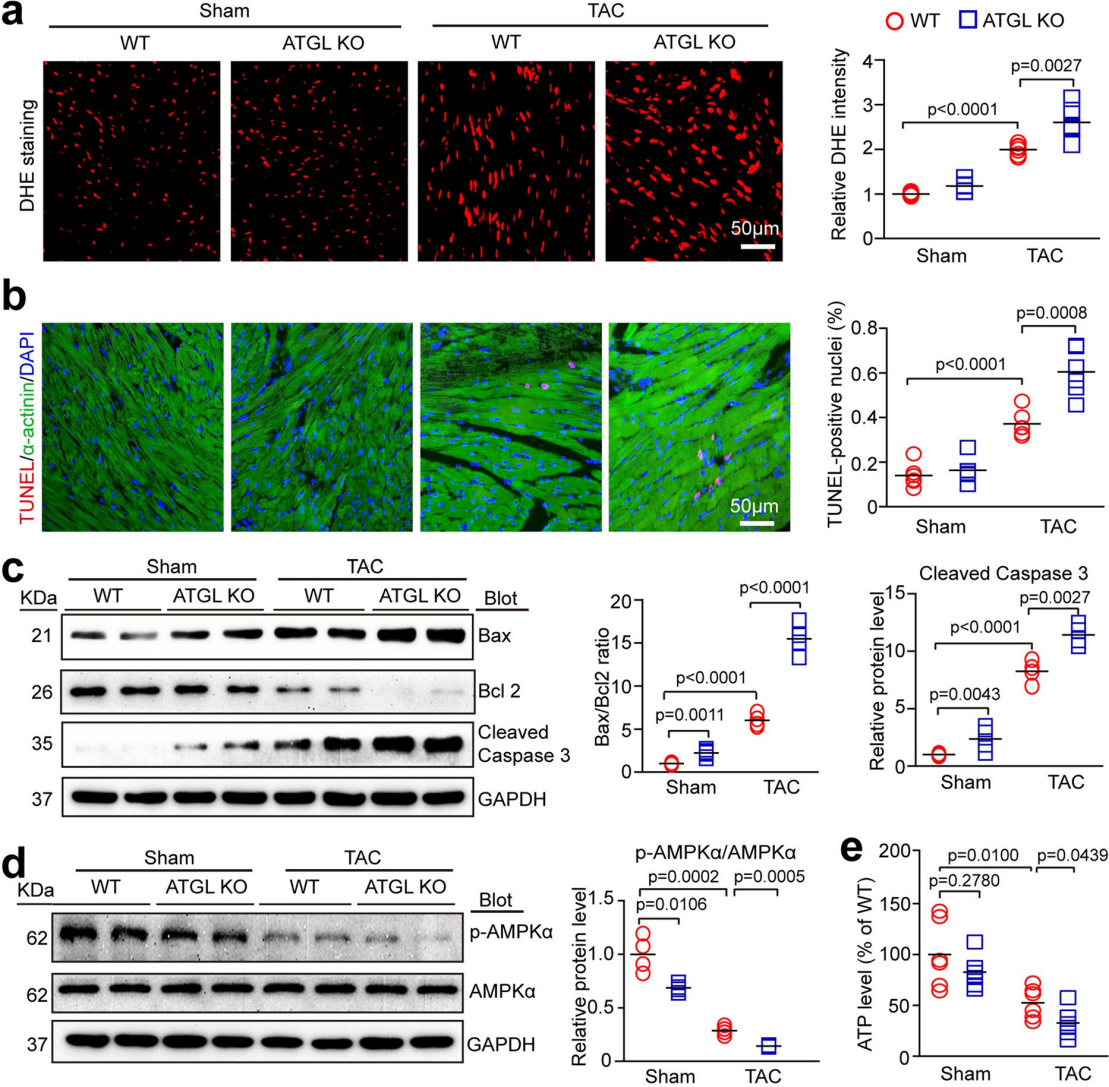
Deficiency of ATGL accelerates pressure overload-induced cardiac oxidative stress and apoptosis. **a**, WT and ATGL KO mice were subjected to sham or TAC operation for 4 weeks. Representative images of dihydroethidium (DHE) staining of the heart sections (left). Quantification of the relative superoxide production (right, *n* = 6). Scale bar: 50 μm. **b**, Representative images of TUNEL (red), α-actinin (green) and DAPI (blue) staining of the heart sections (left), and quantification of TUNEL-positive nuclei (right, *n* = 6). Scale bar: 50 μm. **c**, Representative immunoblotting of Bax, Bcl-2, cleaved capsase-3 and GAPDH in the heart (left). Quantification of the relative protein levels (right, *n* = 4). **d**, Representative immunoblotting of p-AMPKα, AMPKα and GAPDH in the heart (left). Quantification of the relative protein levels (right, *n* = 4). **e**, Measurement of ATP level in the heart tissues. Data are presented as mean ± SEM, and n represents number of animals

We then determined if ATGL KO affects superoxide production and apoptosis, the main causes of myocardial dysfunction. The dihydroethidium (DHE) staining and TUNEL assays indicated that DHE intensities and the percentage of TUNEL-positive cells were significantly higher in TAC-operated WT hearts than in sham-operated controls, and these effects were markedly accelerated in TAC-treated ATGL KO hearts (Fig. 3a–b). Accordingly, the levels of apoptosis-regulating proteins, such as Bax to Bcl-2 ratio and cleaved caspase-3 were also highly enhanced, but levels of p-AMPKα (a key regulator of mitochondrial ATP generation) and ATP production were significantly suppressed in ATGL KO hearts relative to WT hearts following sham or TAC surgery (Fig. 3c–d). However, ATP production levels in ATGL KO hearts and WT hearts were comparable after sham surgery (Fig. 3e).

### ATGL deficiency enhanced TAC-induced inhibition of autophagy

To evaluate the potential mechanisms through which ATGL KO aggravated TAC-induced cardiac dysfunction, we first analyzed the effects of ATGL KO on myocardial lipid deposition. Red oil staining revealed that there were no significant changes in the myocardial lipid levels, including TG content in ATGL KO mice relative to WT controls following sham or TAC surgery (Supplementary Fig. 2a-b). Moreover, the levels of TG, TC, LDL-C, and HDL-C in plasma of ATGL KO and WT mice were comparable (Supplementary Fig. 2c). These results indicate that ATGL KO did not affect cardiac lipid metabolism.

**Fig. 4:**
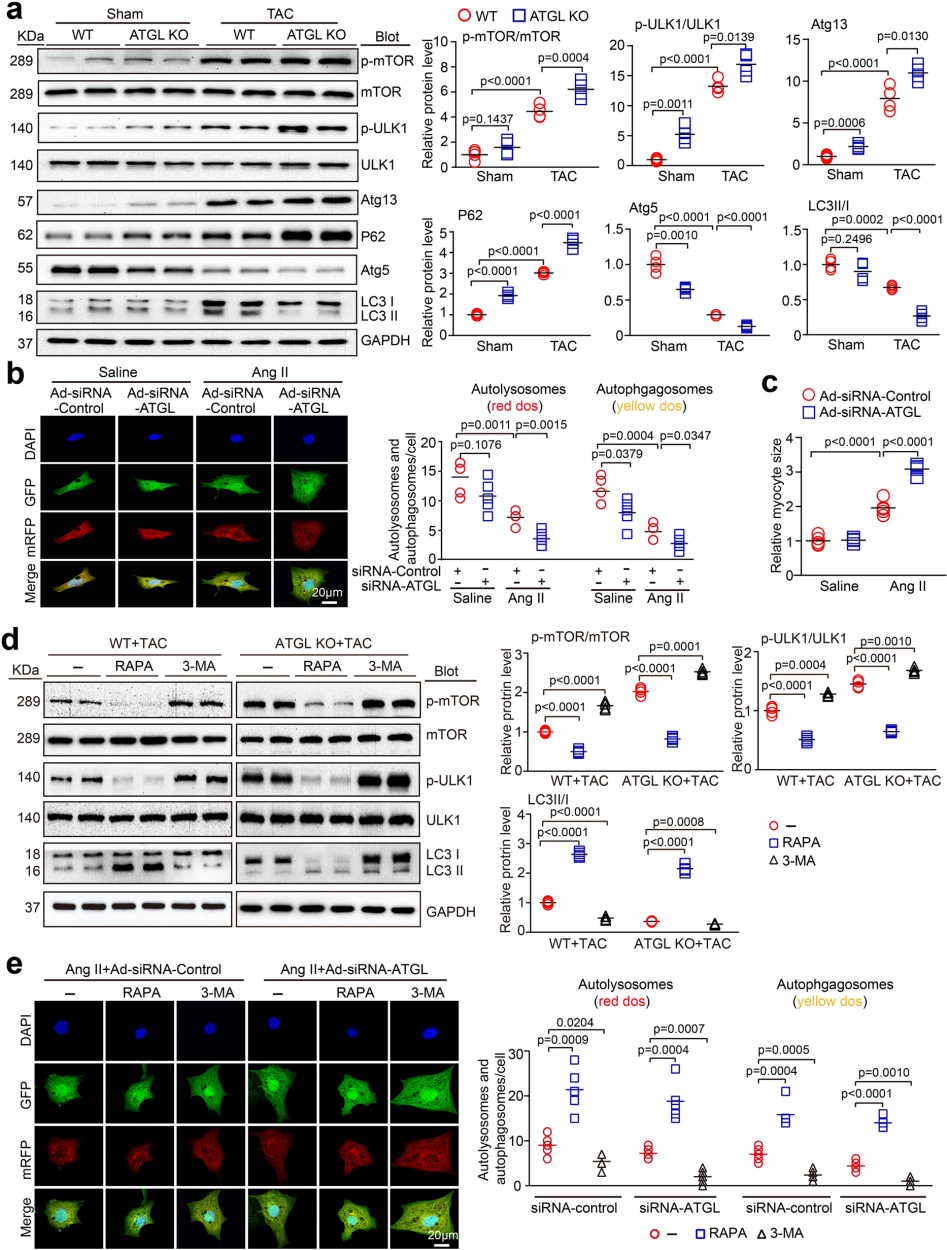
ATGL knockout enhances pressure overload-induced inhibition of autophagic signaling. **a**, WT and ATGL KO mice were subjected to sham or TAC operation for 4 weeks. Representative immunoblotting of p-mTOR, mTOR, p-ULK1, ULK1, Atg13, p62, LC3, Atg5, and GAPDH in the heart (left). Quantification of the relative protein levels (right, *n* = 4). **b**, Neonatal rat cardiomyocytes (NRCMs) were infected with adenovirus containing mRFP-GFP-LC3 together with Ad-siRNA-control or Ad-siRNA-ATGL and then treated with Ang II (100 nM) for 48 h. Immunofluorescence images (right) and the quantification of autophagosomes (yellow) and autolysosomes (red) in each condition (right, 15–20 cells per group) (*n* = 5 independent experiments). Scale bar: 20 μm. **c**, The quantification of cardiomyocyte size in each condition (right, 15–20 cells per group) (*n* = 5 independent experiments). Scale bar: 20 μm. **d**, WT and ATGL KO mice were subjected to TAC and co-treated with rapamycin (RAPA, 4 mg/kg daily) or 3-MA (10 mg/kg daily) for 4 weeks. Representative immunoblotting of p-mTOR, mTOR, p-ULK1, ULK1, and LC3 and GAPDH in the heart (left). Quantification of the relative protein levels (right, *n* = 4). **e**, NRCMs were infected with adenovirus containing mRFP-GFP-LC3 together with Ad-siRNA-control or Ad-siRNA-ATGL and then treated with Ang II (100 nM) in the presence of RAPA (20 nM) or 3-MA (5 mM) for 48 h. Immunofluorescence images (left) and the quantification of autophagosomes (yellow) and autolysosomes (red) in each condition (right, 15–20 cells per group) (*n* = 5 independent experiments). Scale bar: 20 μm. Data are presented as mean ± SEM, and n represents number of animals

Given the role of ATGL in autophagy activation during hepatic lipid metabolism (Martinez-Lopez et al. 2016; Sathyanarayan et al. 2017), we therefore examined autophagy signaling mediators, that are crucial for cardiomyocyte apoptosis and cardiac remodeling. We observed that TAC significantly inactivated autophagy, as indicated by increased protein levels of p-mTOR, p-ULK1, Atg13, and p62 as well as suppressed Atg5 levels and LC3II/I ratios in WT hearts. This inhibitory effect was further enhanced in TAC-treated ATGL KO hearts (Fig. 4a). Interestingly, the protein levels of p-ULK1, Atg13, and p62 were higher while Atg5 level was lower in ATGL KO hearts than in WT hearts after sham surgery (Fig. 4a).

To confirm the effects of ATGL KO on autophagy, we assessed autophagic flux in neonatal rat cardiomyocytes (NRCMs) using tandem fluorescent mRFP-GFP-LC3 along with Ad-siRNA control or Ad-siRNA-ATGL. After 48 h of Ang II treatment, both the number of both autolysosomes (red) and autophagosomes (yellow) were markedly reduced but cardiomyocyte sizes were enhanced in Ad-siRNA-ATGL-infected NRCMs compared with Ad-siRNA-controls (Fig. 4b–c). Similarly, the number of autophagosomes (yellow) was also lower in Ad-siRNA-ATGL cells after saline treatment (Fig. 4b), indicating that ATGL KO reduced autophagic flux.

To determine if autophagy is a downstream target of ATGL, WT or ATGL KO mice were treated with rapamycin (RAPA, the mTOR inhibitor) to activate autophagy or 3-MA to inhibit autophagy after which they were continuously subjected to TAC for 4 weeks. In WT mice, RAPA significantly activated autophagy, as indicated by suppressed levels of p-mTOR, p-ULK1 and elevated LC3II/I ratios compared with vehicle-treated controls. In contrast, 3-MA markedly inhibited autophagic activation (Fig. 4d). Similarly, in TAC-operated ATGL KO mice, the effect of RAPA or 3-MA on autophagy was further agrravated compared with vehicle-treated controls (Fig. 4d).

Then, we confirmed the influence of ATGL KO on autophagy by assessing autophagic flux in NRCMs via infections by the Ad-siRNA-control or Ad-siRNA-ATGL in the presence or absence of RAPA or 3-MA. Compared to vehicle-treated controls, RAPA markedly elevated autophagic flux, as shown by increased numbers of autolysosomes (red) and autophagosomes (yellow) in both Ad-siRNA-control- and Ad-siRNA-ATGL-infected NRCMs. Treatment with 3-MA reversed this effect following Ang II treatment (Fig. 4e). Together, these results suggest that ATGL KO inhibits autophagy.

### Autophagy is involved in ATGL KO-mediated cardiac dysfunction and hypertrophic remodeling

**Fig. 5:**
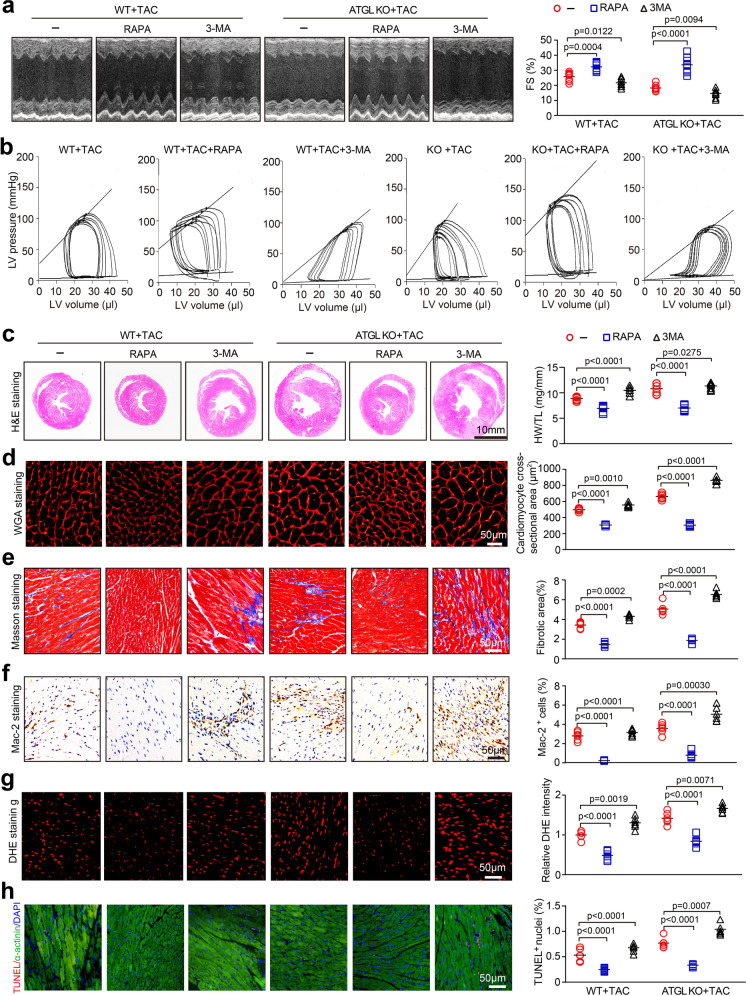
Autophagy mediates pressure overload-induced cardiac hypertrophic remodeling and dysfunction in ATGL KO mice. **a**, WT and ATGL KO mice were subjected to TAC and co-treated with RAPA (4 mg/kg daily) or 3-MA (10 mg/kg daily) for 4 weeks. Echocardiographic measurement of LV chamber (left), and the calculation of LV FS% (right, *n* = 8). **b**, Pressure–volume analysis of systolic and diastolic function. Representative LV P–V loop in each group. **c**, Representative H&E images of heart sections (left). Scale bar: 10 mm. The ratio of HW/TL (right, *n* = 6). **d**, TRITC-labeled WGA staining to measure cardiac myocyte size (left). The Quantification of cross-sectional area of myocytes (right, *n* = 6,150–200 cells counted per sample). Scale bar: 50 μm. **e**, Masson’s trichrome staining of heart sections (left), and quantification of fibrotic area (right, *n* = 6). Scale bar: 50 μm. **f**, Immunohistochemical staining of heart sections with anti-Mac-2 antibody (left). The percentage of Mac-2-positive macrophages (right, *n* = 6). Scale bar: 50 μm. **g**, DHE staining of heart sections (left), and quantification of the relative superoxide production (right, *n* = 6). Scale bar: 50 μm. **h**, Staining of sections for TUNEL (red), α-actinin (green) and DAPI (blue) (left). Quantification of the TUNEL-positive nuclei (right, *n* = 6). Scale bar: 50 μm. Data are presented as mean ± SEM, and n represents number of animals

To determine whether autophagy is involved in ATGL KO-mediated dysfunction and cardiac remodeling, WT or ATGL KO mice were treated with RAPA or 3-MA and then continuously subjected to TAC for 4 weeks. In WT mice, TAC-induced cardiac dysfunction (decreased FS%) was remarkably restored by RAPA, but aggravated by 3-MA (Fig. 5a). The effects of RAPA or 3-MA on TAC-induced suppression of cardiac functions (Alterations of P–V loop, LVSP, SV, EF, Ea, Tau, and − dP/dt) were confirmed by invasive pressure–volume analysis (Fig. 5b; Supplemental Table 4). Accordingly, TAC-triggered cardiac hypertrophy (reduced heart sized, LV wall thickness, HW to TL ratio, and myocyte area) (Fig. 5c–d), interstitial fibrosis (Fig. 5e), infiltration of Mac-2^+^ macrophages (Fig. 5f), superoxide production (Fig. 5g), and the percentage of TUNEL^+^ myocytes (Fig. 5h) were significantly attenuated in RAPA-treated hearts relative to vehicle-treated hearts (Fig. 5c–h). However, these effects were enhanced in 3-MA-treated WT mice (Fig. 5a–h). Moreover, in ATGL KO mice, the effects of RAPA or 3-MA on TAC-triggered cardiac hypertrophy and dysfunction were comparable to WT mice but were further enhanced (Fig. 5a–h; Supplemental Table 4). Therefore, autophagy participated in TAC-induced cardiac remodeling in ATGL KO mice.

### ATGL KO inhibited autophagy via the proteasome-PTEN-mTOR signaling

**Fig. 6:**
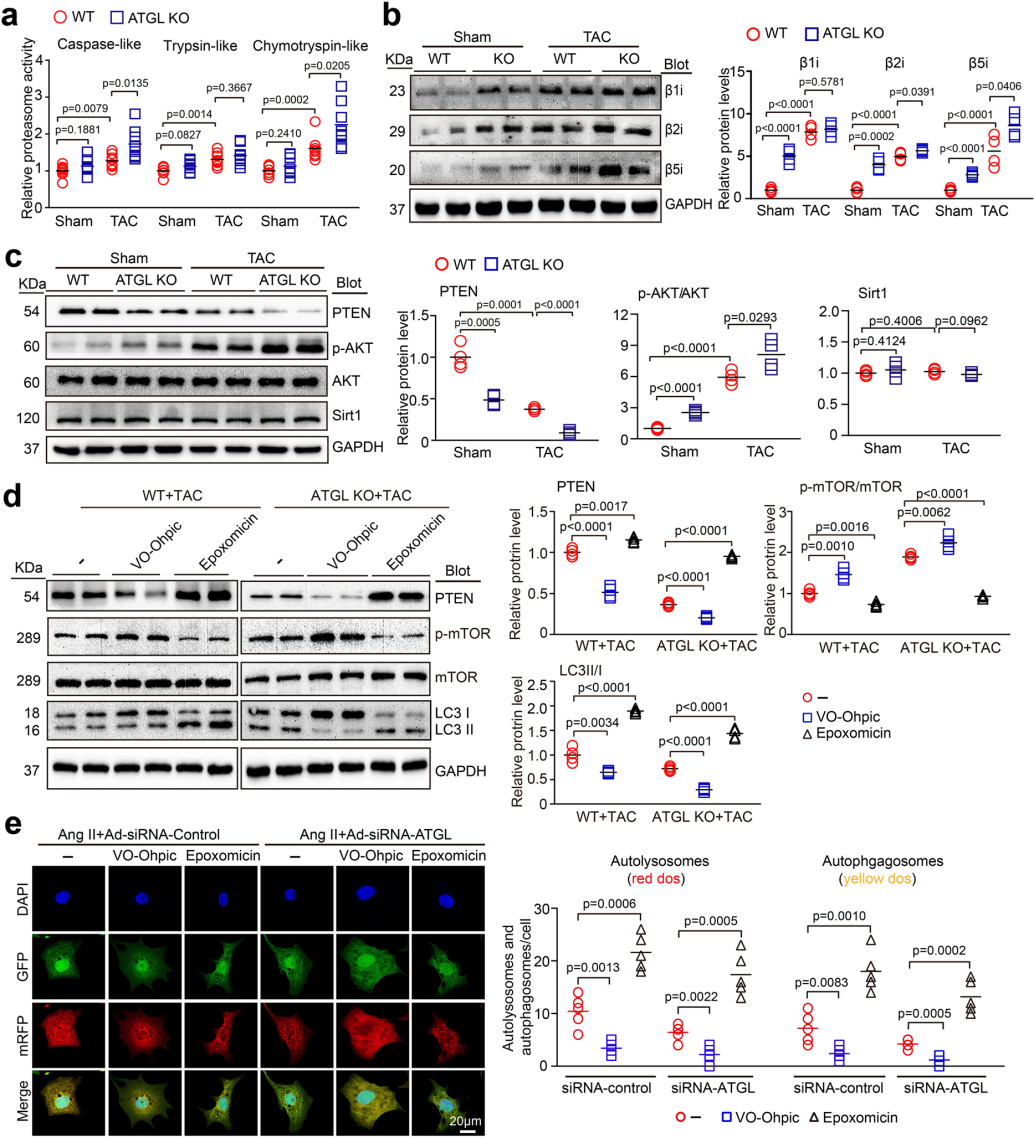
Blocking proteasome activity reverses PTEN stability and activation of autophagy in ATGL KO mice and cardiomyocytes. **a**, WT and ATGL KO mice were subjected to sham or TAC operation for 4 weeks. Measurement of the proteasome caspase-like, trypsin-like, and chymotrypsin-like activities in the heart (*n* = 8). **b**, Immunoblotting of the proteasome catalytic subunits (β1i, β2i, and β5i) in the heart (left), and quantification of the relative protein levels (right, *n* = 4). **c**, Immunoblotting of PTEN, p-AKT, AKT, Sirt1 and GAPDH in the heart (left). Quantification of the relative protein levels (right, *n* = 4). **d**, WT and ATGL KO mice were subjected to TAC operation and co-treated with VO-OHpic (10 mg/kg daily) or epoxomicin (2.9 mg/kg daily) continuously for 4 weeks. Immunoblotting analysis of PTEN, p-mTOR, mTOR, LC3, and GAPDH in the heart (left). Quantification of the relative protein levels (right, *n* = 4). **e**, Neonatal rat cardiomyocytes (NRCMs) were co-infected with adenovirus containing mRFP-GFP-LC3 and Ad-siRNA-control or Ad-siRNA-ATGL, and then treated with Ang II (100 nM) in the presence or absence of VO-OHpic (50 nM) or epoxomicin (100 nM) continuously for 48 h. Immunofluorescence images (left) and the numbers of autophagosomes (yellow) and autolysosomes (red) in each condition (right, 15–20 cells per group) were quantified (*n* = 5 independent experiments). Scale bar: 20 μm. Data are presented as mean ± SEM, and *n* represents number of animals

The proteasome complex is highly induced by various hypertrophic stimuli and is crucial for regulating autophagy and PTEN stability in the hearts (Cao et al. 2019; Chen et al. 2019; Han et al. 2020; Xie et al. 2019; Xie et al. 2020; Yan et al. 2020). Therefore, we assessed the effect the of ATGL KO on the catalytic subunit expressions and activities in the hearts. Relative to sham controls, TAC surgery markedly upregulated the proteasome activities (chymotrypsin-like, trypsin-like, and caspase-like) and the protein levels of immunosubunits (β1i, β2i, and β5i), and this effect was further enhanced in ATGL KO mice following TAC treatment (Fig. 6a–b). Furthermore, TAC-induced downregulation of PTEN (a negative regulator of AKT/mTOR signaling) and upregulation of p-AKT in WT mice were also accelerated in ATGL KO mice (Fig. 6c), suggesting that the proteasome may be involved in the degradation of PTEN protein. Interestingly, the protein levels of Sirt1 (Sirtuin 1, an autophagy activator) (Lee et al. 2008) were not significant changed between in ATGL KO and WT hearts following sham or TAC surgery (Fig. 6c).

To define whether the proteasome regulates PTEN degradation and mTOR-mediated autophagy in vivo, we treated WT or ATGL KO mice with epoxomicin (a proteasome inhibitor) or VO-OHpic (a specific PTEN inhibitor) and then subject them to TAC continuously for 4 weeks. In both WT and ATGL KO mice, TAC-induced upregulation of proteasomal caspase-like, trypsin-like, and chymotrypsin-like activities were significantly suppressed by epoxomicin but enhanced by VO-OHpic treatments (Supplementary Fig. 3). Moreover, VO-OHpic treatment markedly suppressed PTEN and LC3 II/I ratios, but enhanced p-mTOR levels, and these effects were highly reversed by epoxomicin treatment in TAC-operated WT hearts (Fig. 6d). Accordingly, after Ang II treatment, VO-OHpic also markedly inhibited the autophagic flux, as indicated by decreased autolysosomes (red) and autophagosomes (yellow) in both Ad-siRNA-infected NRCMs. Conversely, epoxomicin remarkably upregulated autophagic flux (Fig. 6e). Meanwhile, the effect of VO-OHpic or epoxomicin on PTEN-mTOR-LC3 II/I signals as well as autophagic flux were further enhanced in TAC-treated ATGL KO mice (Fig. 6d, e). Collectively, these findings imply that ATGL KO inhibited autophagy by enhancing PTEN proteasomal degradation, leading to activation of autophagy in TAC-induced hypertrophic hearts.

### Blockage of the proteasome activity attenuated TAC-induced cardiac remodeling in ATGL KO mice

**Fig. 7:**
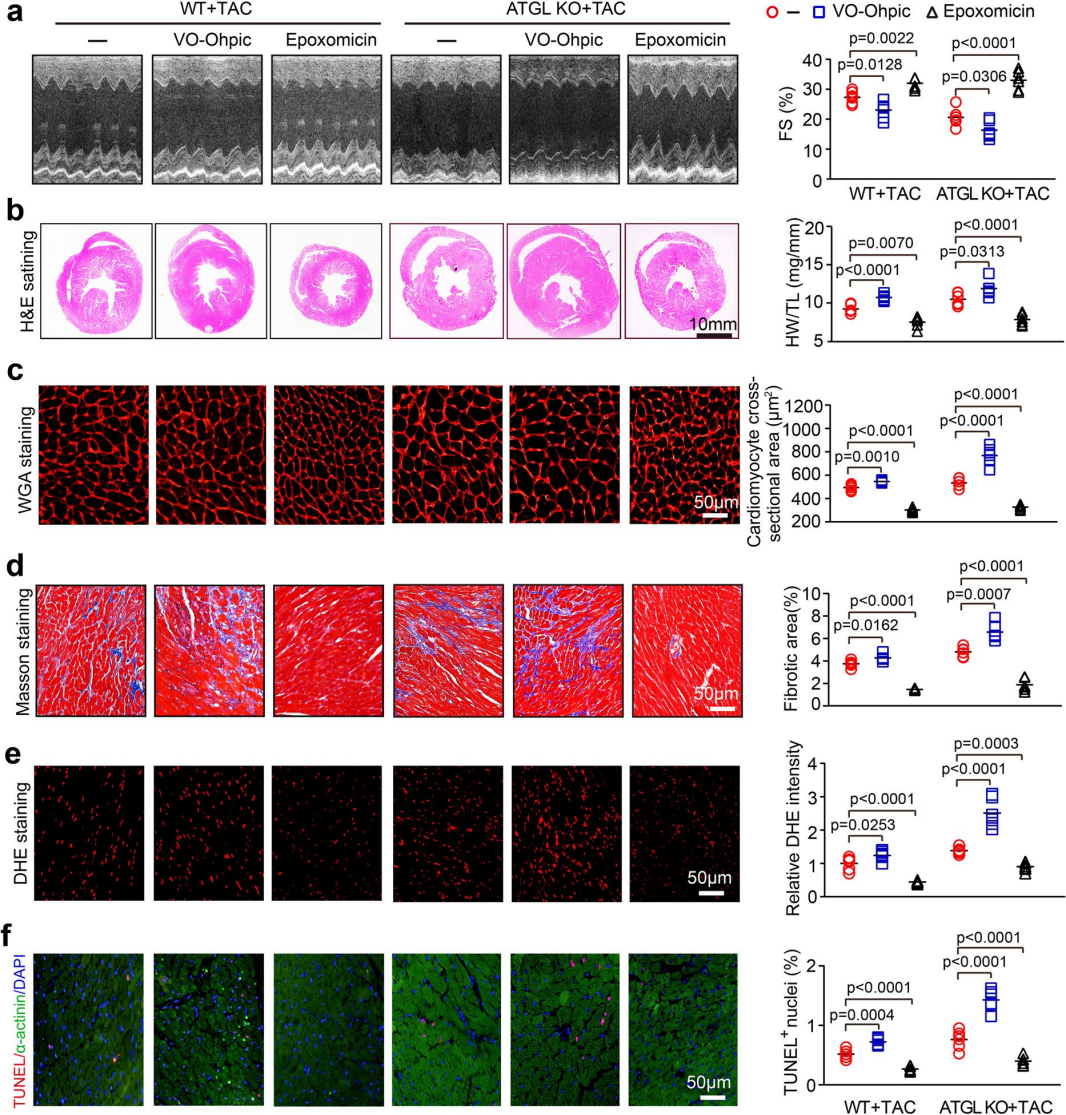
Blocking proteasome activity reverses TAC-induced cardiac dysfunction and hypertrophic remodeling. **a**, WT and ATGL KO mice were subjected to TAC operation and co-treated with VO-OHpic (10 mg/kg daily) or epoxomicin (2.9 mg/kg daily) continuously for 4 weeks. Echocardiographic measurement of LV chamber (left), and the calculation of LV FS% (right, *n* = 6). **b**, Representative H&E staining of heart sections (left). Scale bar: 10 mm. The ratio of HW/TL (right, *n* = 6). **c**, TRITC-labeled WGA staining of heat sections (left). Quantification of cross-sectional area of myocytes (right, *n* = 6, 150–200 cells counted per sample). Scale bar: 50 μm. **d**, Masson’s trichrome staining of heat sections (left). Quantification of fibrotic area (right, *n* = 6). Scale bar: 50 μm. **e**, DHE staining of heart sections (left). Quantification of the relative superoxide production (right, *n* = 6). Scale bar: 50 μm. **f**, TUNEL staining (left). Quantification of the TUNEL-positive nuclei (right, *n* = 6). Scale bar: 50 μm. Data are presented as mean ± SEM, and *n* represents number of animals

To test whether the proteasome is involved in TAC-induced cardiac dysfunction and remodeling, WT or ATGL KO mice were administrated with epoxomicin ot VO-OHpic. After 4 weeks of TAC surgery, TAC-induced suppression of cardiac functions (decreased FS%) (Fig. 7a), and enhancement of cardiac hypertrophy (increased LV wall thickness, ratios of HW/TL, and myocyte area) (Fig. 7b–c), fibrosis (Fig. 7d), superoxide production (Fig. 7e), and myocyte apoptosis (Fig. 7f) in vehicle-treated WT hearts were further aggravated in VO-OHpic-administered ATGL KO mice (Fig. 7a–f). However, these deleterious effects were restored in epoxomicin-administered WT mice (Fig. 7a–f). Moreover, the effects of epoxomicin or VO-OHpic on cardiac dysfunction and remodeling were further enhanced in TAC-operated ATGL mice (Fig. 7a–f), demonstrating that ATGL KO accelerates cardiac hypertrophic remodeling by enhancing PTEN degradation by the proteasome.

## Discussion

In this study, we provide novel evidence that ATGL exerts a cardioprotective role in response to pressure overload. ATGL expression was markedly decreased in TAC-induced hypertrophic heart. ATGL KO significantly promoted TAC-induced cardiac hypertrophy and dysfunction accompanied with reduction of PTEN protein and activation of AKT-mTOR signaling as well as inhibition of autophagy. These effects were reversed by proteasome inhibitor (epoxomicin) or autophagic activator (RAPA) but accelerated by PTEN inhibitor (VO-OHpic) or autophagy inhibitor 3-MA. Mechanistically, ATGL KO upregulates proteasome subunit expression and activities, which then mediates PTEN degradation leading to activation AKT-mTOR signaling and inhibition of autophagy, thereby promoting hypertrophic remodeling and dysfunction. Overall, our results suggest that ATGL acts as a novel regulator of cardiac hypertrophic remodeling probably associated with the proteasome-PTEN-mTOR-autophagy pathway.

ATGL is the rate-limiting enzyme in lipolysis and is predominantly expressed in adipose tissues and in other tissues, such as heart (Kintscher et al. 2020). ATGL plays a critical role in modulating lipid metabolism and cardiac function (Foryst-Ludwig et al. 2015; Salatzki et al. 2018). Ablation of ATGL in adipose tissues or inhibition of ATGL by Atglistatin (predominantly targeting ATGL in adipose tissue) was shown to prevent cardiac damage and dysfunction by reducing galectin-3 or fatty acid secretion from adipocyte tissues (Takahara et al. 2021; Thiele et al. 2021), implying that adipocytic ATGL is involved in cardiac remodeling and dysfunctions. However, several studies have revealed that ATGL in cardiomyocytes directly regulates cardiac metabolism and mitochondrial substrate oxidation through different mechanisms (Gao et al. 2015, Haemmerle et al. 2011; Kienesberger et al. 2012). Interestingly, ATGL deficiency exhibited age-dependent upregulation of TG levels in cardiomyocytes, with starting at 6 weeks of age (Haemmerle et al. 2006). However, our results show that ATGL KO in mice at 12 weeks of age had no significant effects on cardiac TG and plasma lipid levels under basal conditions or following 4 weeks of TAC surgery (Supplementary Fig. 2). These differences in outcomes have not been elucidated; therefore, we determined if other mechanisms, such as autophagy, are involved in pressure overload-induced cardiac hypertrophic remodeling.

Autophagy is a degradative process that promotes lysosomal degradation of proteins and organelles, which is regulated by autophagy-related genes, including ULK1, Atg13, Atg5, Atg6, and Atg8/LC3 (Wang and Cui 2017). The ULK1/Atg13 complex is the most upstream mediator of autophagic induction and is phosphorylated by mTOR (Ganley et al. 2009). Autophagic dysregulation has been shown to affect cardiomyocyte apoptosis and function, leading to cardiac hypertrophy (Li et al. 2016). Overexpressions of Atg6 in cardiomyocytes aggravates pressure overload-induced HF (Zhu et al. 2007). Conversely, cardiac-specific Atg5 KO enhances TCA-triggered LV hypertrophy and reduced contractile function (Nakai et al. 2007). Here, we found that ATGL KO significantly aggravated TAC-induced activation of apoptosis and inhibition of autophagy accompanied with increased Bax/Bcl-2 ratios and the protein levels of cleaved caspase-3, p-mTOR, p-ULk1, Atg13 and p62, as well as decreased Atg5 protein levels and LC3II/I ratio (Fig. 3c, Fig. 4a–b). Respective activation or inhibition of autophagy in ATGL KO or WT mice using RAPA or 3-MA confirmed that ATGL KO accelerated TAC induced cardiac hypertrophy and LV dysfunction through suppressing autophagy (Fig. 4–5). However, ATGL KO also markedly upregulated Bax/Bcl-2 ratios, cleaved caspase-3, p-ULk1, Atg13, and p62 levels, while downregulated Atg5 levels (Fig. 3c, Fig. 4a), but had no significant effects on cardiac function, hypertrophy, and myocyte apoptosis compared to WT controls (Figs. 1, 2, 3), These finding suggest that ATGL KO-mediated activation of apoptosis- and autophagy-related signals lead to cardiac dysfunction during pressure overload, but not under basal condition.

Previous studies indicated that ATGL stimulates autophagy/lipophagy by interacting with LC3 or by enhancing Sirt1 activity in the liver or adipose tissues (Martinez-Lopez et al. 2016; Salatzki et al. 2018). Sirt1 activates autophagy through deacetylation and activation of multiple key autophagy genes (Atg5, Atg7, and Atg8) (Lee et al. 2008). However, our results indicated that ATGL KO did not significantly affect Sirt1 protein level in the heart tissues after sham or TAC surgery (Fig. 6c), suggesting that Sirt1 does not participate in inhibition of autophagy in ATGL KO hearts. Interestingly, PTEN/AKT/mTOR signaling critically regulates autophagy via ULK1 phosphorylation (Kim et al. 2018; Shi et al. 2020). PTEN is a key negative regulator of AKT-mTOR and AMPK signaling pathways, which exerts a critical role in the regulation of myocardial hypertrophic remodeling. Its stability is mainly regulated by the proteasome catalytic subunits especially β1i, β2i and β5i (Chen et al. 2019, Li et al. 2018; Xu et al. 2014; Zou et al. 2019). In this study, ATGL KO markedly enhanced the expressions and activities of the proteasome catalytic subunits and PTEN degradation (Fig. 6a–c). Conversely, treatment of ATGL KO mice with epoxomicin significantly upregulated PTEN protein level while suppressing AKT/mTOR signaling, leading to autophagic activation and attenuation of cardiac hypertrophic remodeling after TAC operation. However, these actions were markedly reversed by the PTEN inhibitor, VO-OHpic (Fig. 6d–e, Fig. 7). These findings indicate that ATGL KO inhibits autophagy likely through the proteasome-PTEN-mTOR signaling pathway.

Cardiac oxidative stress, inflammation, and apoptosis are involved in hypertrophy as well as HF pathogeneses. In cardiomyocytes, ATGL has been shown to suppress cardiac NADPH oxidase activities, accompanied by downregulation of NADPH oxidase isoforms and pro-inflammatory mediators. These effects are probably due to reduced cardiac PPARα-mediated regulation of antioxidant enzymes and anti-inflammatory effects in ATGL KO mice (Haemmerle et al. 2011; Schrammel et al. 2013; Vegliante et al. 2018). Altered lipid droplets containing cell-type specific surface-binding proteins such as perilipin 5 have been shown to induce oxidative and inflammatory responses (Kuramoto et al. 2012). Interestingly, gp91ds-tat (a selective NADPH oxidase inhibitor) and MnTBAP (antioxidant SOD mimetic) ameliorated oxidative stress in WT and ATGL KO heart homogenates. However, due to insufficient reactivity, quality, or composition, systemic treatment with MnTBAP aggravates cardiac oxidative stress in ATGL KO mice (Batinic-Haberle et al. 2009; Reboucas et al. 2008; Schrammel et al. 2013). Studies should investigate the protective effects of NADPH oxidase inhibitors such as gp91ds-tat or antioxidants such as apocynin in oxidative stress in ATGL KO mice (Liu et al. 2010). Global ATGL KO or ATGL inhibition with atglistatin attenuates AMPK phosphorylation in adipose tissues (MacPherson et al. 2016). Consistently, we found that ATGL KO suppressed AMPK activation in the heart following TAC or sham operation (Fig. 3d). However, there are several limitations in the presents study: The precise mechanisms through which ATGL regulates the activation of AMPK, NF-κB, and NOX signals, the expression and activity of the proteasome catalytic subunits in the heart should be evaluated further. Moreover, ATGL regulates autophagy in cardiomyocyte-specific ATGL knockout mice should be investigated.

In conclusion, our results reveal a novel mechanism for ATGL to prevent pressure overload-induced cardiac hypertrophy and HF. ATGL KO increases the proteasome level and activity, which promotes PTEN degradation and activation of AKT-mTOR signaling, resulting in suppression of autophagy and cardiac hypertrophy and HF.

### Supplementary Information

Below is the link to the electronic supplementary material.

Supplementary file1 (DOCX 24 KB)

Supplementary file2 (DOCX 14 KB)

Supplementary file3 (JPG 507 KB)

Supplementary file4 (JPG 925 KB)

Supplementary file5 (JPG 428 KB)

## Data Availability

The datasets generated during and/or analyzed during the current study are available from the corresponding author on reasonable request.

## Abbreviations

ATGL: Adipose triglyceride lipase
TAC: Transverse aortic constriction
TAG: Triacylglycerol
EF: Left ventricle ejection fraction
FS: Left ventricle fractional shortening
LVSP: Left ventricle systolic pressure
SV: Stroke volume
Ea: Arterial elastance (measure of ventricular afterload)
− dp/dt: Maximal rate of pressure decline (diastolic indexes).
Tau: Relaxation time constant
