# Supplementary material for: ATGL deficiency aggravates pressure overload-triggered myocardial hypertrophic remodeling associated with the proteasome-PTEN-mTOR-autophagy pathway
Source: Cell Biol Toxicol. 2022 Feb 26;39(5):2113–31. doi: 10.1007/s10565-022-09699-0 (PMC10547847; doi:10.1007/s10565-022-09699-0)
Supplement: Supplementary file 1 — Supplementary file1 (DOCX 24 KB) [file 10565_2022_9699_MOESM1_ESM.docx]

**Table S1: primer sequences for qPCR analysis**

| Gene symbol | Forward primer (5’-3’) | Reverse primer (5’-3’) |
| --- | --- | --- |
| IL-1β | CTTCCCCAGGGCATGTTAAG | ACCCTGAGCGACCTGTCTTG |
| IL-6 | TTCCATCCAGTTGCCTTCTTG | TTGGGAGTGGTATCCTCTGTGA |
| ANF | CACAGATCTGATGGATTTCAAGA | CCTCATCTTCTACCGGCATC |
| BNP | GAAGGTGCTGTCCCAGATGA | CCAGCAGCTGCATCTTGAAT |
| Collagen I | GAGTACTGGATCGACCCTAACCA | GACGGCTGAGTAGGGAACACA |
| Collagen III | TCCCCTGGAATCTGTGAATC | TGAGTCGAATTGGGGAGAAT |
| GAPDH | GGTTGTCTCCTGCGACTTCA | GGTGGTCCAGGGTTTCTTACTC |

**Table S2: Primary antibodies used in immunoblotting analysis**

| **Antibody** | **Species** | **Dilution** | **Company** |
| --- | --- | --- | --- |
| anti-PTEN | human, mouse, rat | 1:2000 | Cell Signaling Technology (#9188) |
| anti-p-mTOR | human, mouse, rat | 1:2000 | Cell Signaling Technology (#5536) |
| anti-mTOR | human, mouse, rat | 1:2000 | Cell Signaling Technology (#2972) |
| anti-p-AKT | human, mouse, rat | 1:2000 | Cell Signaling Technology (#9271) |
| anti-AKT | human, mouse, rat | 1:2000 | Cell Signaling Technology (#9272) |
| anti-p-ERK1/2 | human, mouse, rat | 1:2000 | Cell Signaling Technology (#4370) |
| anti-ERK1/2 | human, mouse, rat | 1:2000 | Cell Signaling Technology (#4695) |
| anti-ATGL | human, mouse, rat | 1:2000 | Cell Signaling Technology (#2138) |
| TGF-β1 | human, mouse, rat | 1:1000 | Cell Signaling Technology (#3711s) |
| anti-p-NF-κB | human, mouse, rat | 1:1000 | Cell Signaling Technology (#3033) |
| anti- NF-κB | human, mouse, rat | 1:3000 | Cell Signaling Technology (#4764) |
| anti-p-AMPKα | human, mouse, rat | 1:1000 | Cell Signaling Technology (#2535) |
| anti-AMPKα | human, mouse, rat | 1:3000 | Cell Signaling Technology (#2532) |
| anti-p-ULK1 | human, mouse, rat | 1:1000 | Cell Signaling Technology (#14202) |
| anti-ULK1 | human, mouse, rat | 1:3000 | Cell Signaling Technology (#8054) |
| anti-LC3B | human, mouse, rat | 1:1000 | Cell Signaling Technology (#43566) |
| anti-SirT1 | human, mouse, rat | 1:1000 | Cell Signaling Technology (#2028) |
| anti-Atg5 | human, mouse, rat | 1:1000 | Cell Signaling Technology (#12994) |
| GAPDH | human, mouse, rat | 1:5000 | Cell Signaling Technology (#2118) |
| Anti-β1i | human, mouse, rat | 1:1000 | Abcam (ab203224) |
| anti-β2i | human, mouse, rat | 1:1000 | Abcam (ab183506) |
| anti- β5i | human, mouse, rat | 1:1000 | Abcam (ab3329) |
| anti-Atg13 | human, mouse, rat | 1:1000 | Abcam (ab105392) |
| anti-p62 | human, mouse, rat | 1:1000 | Abcam (ab240635) |
| Bax | human, mouse, rat | 1:2000 | Proteintech (50599-2-Ig) |
| Bcl-2 | human, mouse, rat | 1:2000 | Proteintech (26593-1-AP) |
| Caspase-3 | human, mouse, rat | 1:2000 | Proteintech (19677-1-AP) |

**Table S3: Cardiac pressure-volume loop analysis in wild-type (WT) and ATGL- KO mice after 4 weeks of TAC surgery**

| Parameter | Sham+WT  （n=6） | Sham+ATGL- KO（n=6） | Sham+ATGL- KO（n=6） | ATGL- KO+TAC  （n=6） |
| --- | --- | --- | --- | --- |
| LVSP (mmHg) | 134.33 ± 2.57 | 128.25 ± 2.44 | 103.66 ± 2.58**** | 90.35 ± 0.83^###^ |
| SV (μL) | 11.24 ± 0.31 | 9.91± 0.24 | 7.15 ± 0.34**** | 5.11 ± 0.15^###^ |
| Ea (mmHg/μL) | 17.32 ± 0.49 | 18.42 ± 0.40 | 12.03 ± 0.41**** | 9.37 ± 0.38^###^ |
| Tau (ms) | 10.00 ± 0.41 | 10.83 ± 0.38 | 14.20 ± 0.36**** | 15.92 ± 0.38^##^ |
| -dp/dt (mmHg/s) | -8495.67 ± 246.06 | -8453.33 ± 161.62 | -4310.50 ± 150.02**** | -3193.67 ± 80.73^####^ |
| **+**dp/dt (mmHg/s) | 10248.83 ± 355.14 | 9416.33 ± 534.32 | 6151.50 ± 43.64  **** | 5185.33 ± 52.82^####^ |
| EF (%) | 60.11 ± 1.29 | 60.23 ± 1.40 | 42.89 ± 1.05**** | 33.81 ± 1.61^###^ |

LVSP, LV systolic pressure; SV, stroke volume; EF, ejection fraction; Ea, arterial elastance; Tau, relaxation time constant; -dP/dt, maximal rate of pressure decline. Data are expressed as mean ± SEM. *****P* < 0.0001 versus sham ; ^##^*P* < 0.01; ^###^*P* < 0.001, ^####^*P* < 0.0001 versus WT+TAC.

**Table S4: Cardiac pressure-volume loop analysis in wild-type (WT) and ATGL- KO mice in the presence or absence of RAPA or 3MA after 4 weeks of TAC surgery**

| Parameter | WT+TAC  （n=6） | WT+RAPA  （n=6） | WT+3MA  （n=6） | ATGLKO+TAC  （n=6） | ATGLKO+TAC+RAPA  （n=6） | ATGLKO+TAC+3MA  （n=6） |
| --- | --- | --- | --- | --- | --- | --- |
| LVSP (mmHg) | 113.78±0.99 | 123.67±1.70*** | 99.14±2.48^$$$^ | 90.92±0.91 | 143.48±2.32^####^ | 77.28±1.64^&&&&^ |
| SV (μL) | 6.78±0.33 | 11.33±0.36**** | 5.43±0.22^$$^ | 4.04±0.28 | 11.04±0.19^####^ | 2.99±0.15^&&&^ |
| Ea (mmHg/μL) | 10.06±0.42 | 17.41±0.71**** | 8.70±0.25^$^ | 7.15±0.15 | 17.04±0.73^####^ | 4.11±0.10^&&&&^ |
| Tau (ms) | 13.99±0.48 | 4.67±0.11**** | 16.92±0.87^$^ | 16.79±0.79 | 7.61±0.49^####^ | 25.08±1.29^&&&^ |
| -dp/dt (mmHg/s) | -4525.67±243.98 | -8505.83±145.85**** | -3593.00±260.09^$^ | -3214.00±138.54 | -8345.33±68.38^####^ | -2270.67±24.88^&&&&^ |
| **+**dp/dt (mmHg/s) | 5941±71.51 | 8230.83±101.30**** | 5307.00±139.27^$$^ | 5024.00±33.08 | 7866.33±200.30^####^ | 4038.00±195.66^&&&&^ |
| EF (%) | 41.72±2.08 | 60.60±1.36**** | 35.18±1.99^$^ | 34.08±1.50 | 59.42±2.16^####^ | 21.35±1.17^&&&&^ |

LVSP, LV systolic pressure; SV, stroke volume; EF, ejection fraction; Ea, arterial elastance; Tau, relaxation time constant; -dP/dt, maximal rate of pressure decline. Data are expressed as mean ± SEM. ****P* < 0.001; *****P* < 0.0001 versus WT+TAC ;^$^*P* < 0.05; ^$$^*P* < 0.01; ^$$$^*P* < 0.0001versus WT+TAC ;^####^*P* < 0.0001 versus ATGL- KO+TAC; ^&&&^*P* < 0.001, ^&&&&^*P* < 0.0001 versus ATGL- KO+TAC.
