# Supplementary material for: ATGL deficiency aggravates pressure overload-triggered myocardial hypertrophic remodeling associated with the proteasome-PTEN-mTOR-autophagy pathway
Source: Cell Biol Toxicol. 2022 Feb 26;39(5):2113–31. doi: 10.1007/s10565-022-09699-0 (PMC10547847; doi:10.1007/s10565-022-09699-0)
Supplement: Supplementary file 2 — Supplementary file2 (DOCX 14 KB) [file 10565_2022_9699_MOESM2_ESM.docx]

**Supplementary data**

**Supplementary Figure 1. The dynamic changes of** **cardiac function in WT and ATGL KO mice after TAC opreation.** Wild-type (WT) and ATGL knockout (KO) mice were subjected to sham or transverse aortic constriction (TAC) operation for 2-4 weeks. **a**, Representative blood velocity measurement of aortic arch, the calculation of trans-aortic pressure gradient (n=8). **b**, The calculation of LV ejection fraction (EF%) and fractional shortening (FS%) at different time points after TAC surgery (n=8). Data are expressed as mean ± SEM, and n represents the number of samples. **P* < 0.05; ***P* < 0.005; ****P* < 0.001; *****P* < 0.0001 versus WT control, ^#^*P* < 0.05; ^##^*P* < 0.005; ^##^*P* < 0.001; ^####^*P* < 0.0001 versus versus ATGL KO control, ^&^*P* < 0.05; ^&&^*P* < 0.005; ^&&&^*P* < 0.001; ^&&&&^*P* < 0.0001 versus versus WT TAC.

**Supplementary Figure 2. ATGL deficiency had no effect on heart lipid content and TG levels after TAC operation.** WT and ATGL KO mice were subjected to sham or transverse aortic constriction (TAC) operation for 4 weeks. **a**, Representative images of Red oil staining of the heart sections (n=6). Scale bar: 50 μm. **b**, ELISA analysis of myocardial TG level (n=5). **c**, ELISA measurement of plasma TG, total cholesterol (TC), low density lipoprotein cholesterol (LDL-C), and high-density lipoprotein cholesterol (HDL-C) (n=8). Data are expressed as mean ± SEM, and n represents the number of samples.

**Supplementary Figure 3. The effect of VO-OHpic or Epoxomicin on the proteasome activity in ATGL KO mice after TAC operation.** WT and ATGL KO mice were subjected to TAC operation and co-treated with VO-OHpic (10 mg/kg daily) or Epoxomicin (2.9 mg/kg daily) continuously for 4 weeks. Measurement of the proteasome caspase-like, trypsin-like, and chymotrypsin-like activities in the heart (n=6). Data are presented as mean ± SEM, and n represents number of animals.
