# Supplementary figures and images for: ATGL deficiency aggravates pressure overload-triggered myocardial hypertrophic remodeling associated with the proteasome-PTEN-mTOR-autophagy pathway
Source: Cell Biol Toxicol. 2022 Feb 26;39(5):2113–31. doi: 10.1007/s10565-022-09699-0 (PMC10547847; doi:10.1007/s10565-022-09699-0)

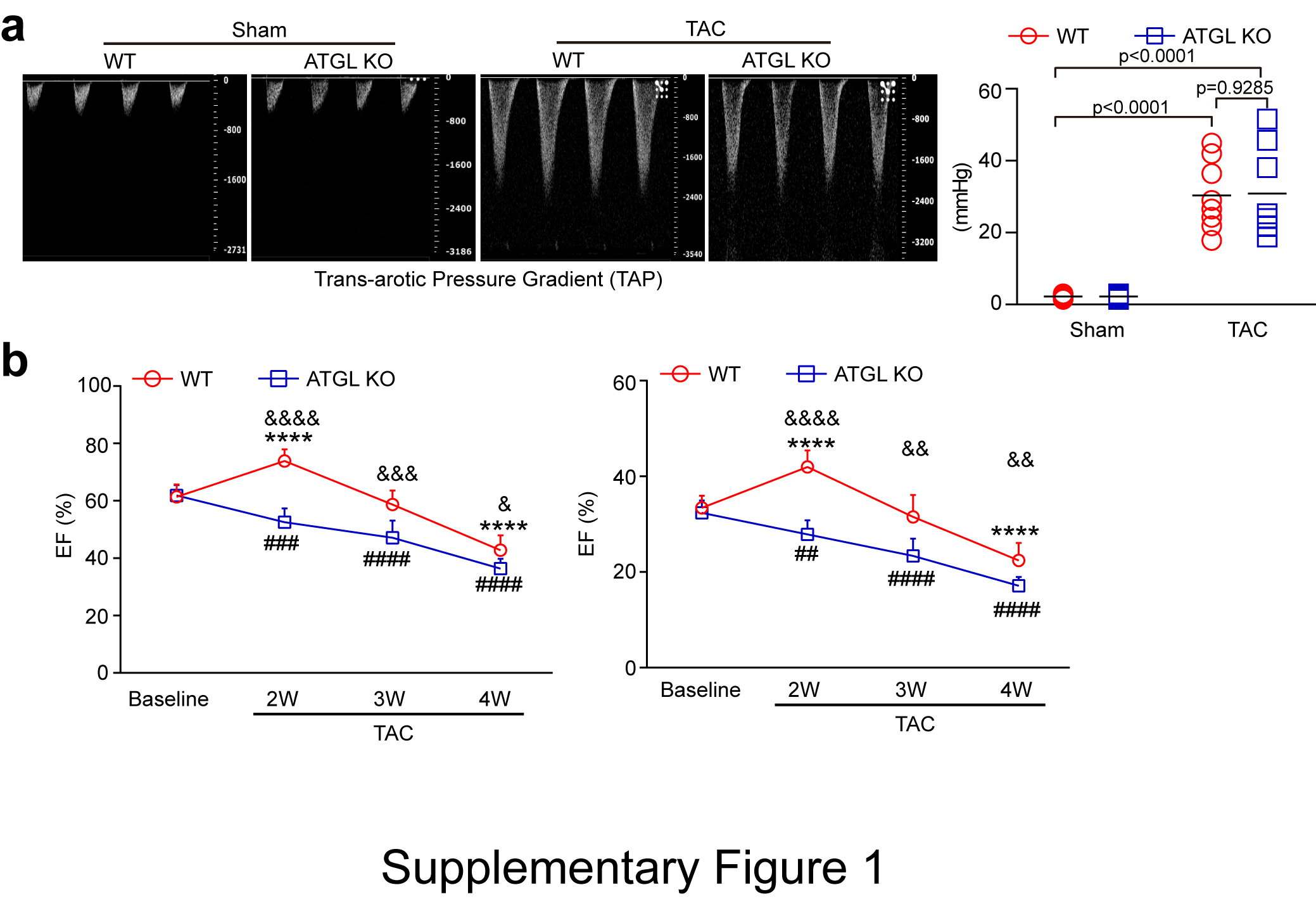

Supplement: Supplementary file 3 — Supplementary file3 (JPG 507 KB) [file 10565_2022_9699_MOESM3_ESM.jpg]

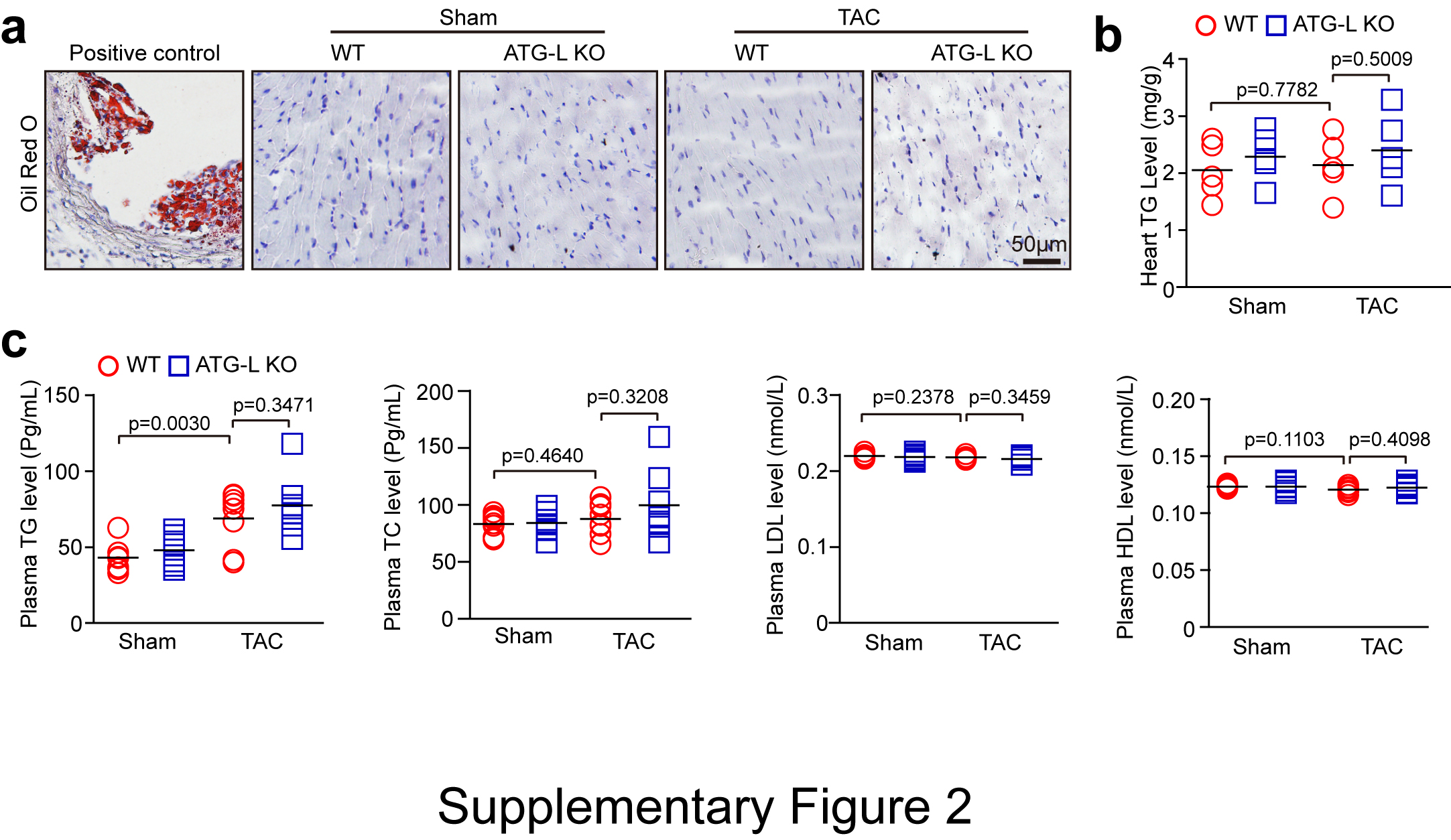

Supplement: Supplementary file 4 — Supplementary file4 (JPG 925 KB) [file 10565_2022_9699_MOESM4_ESM.jpg]

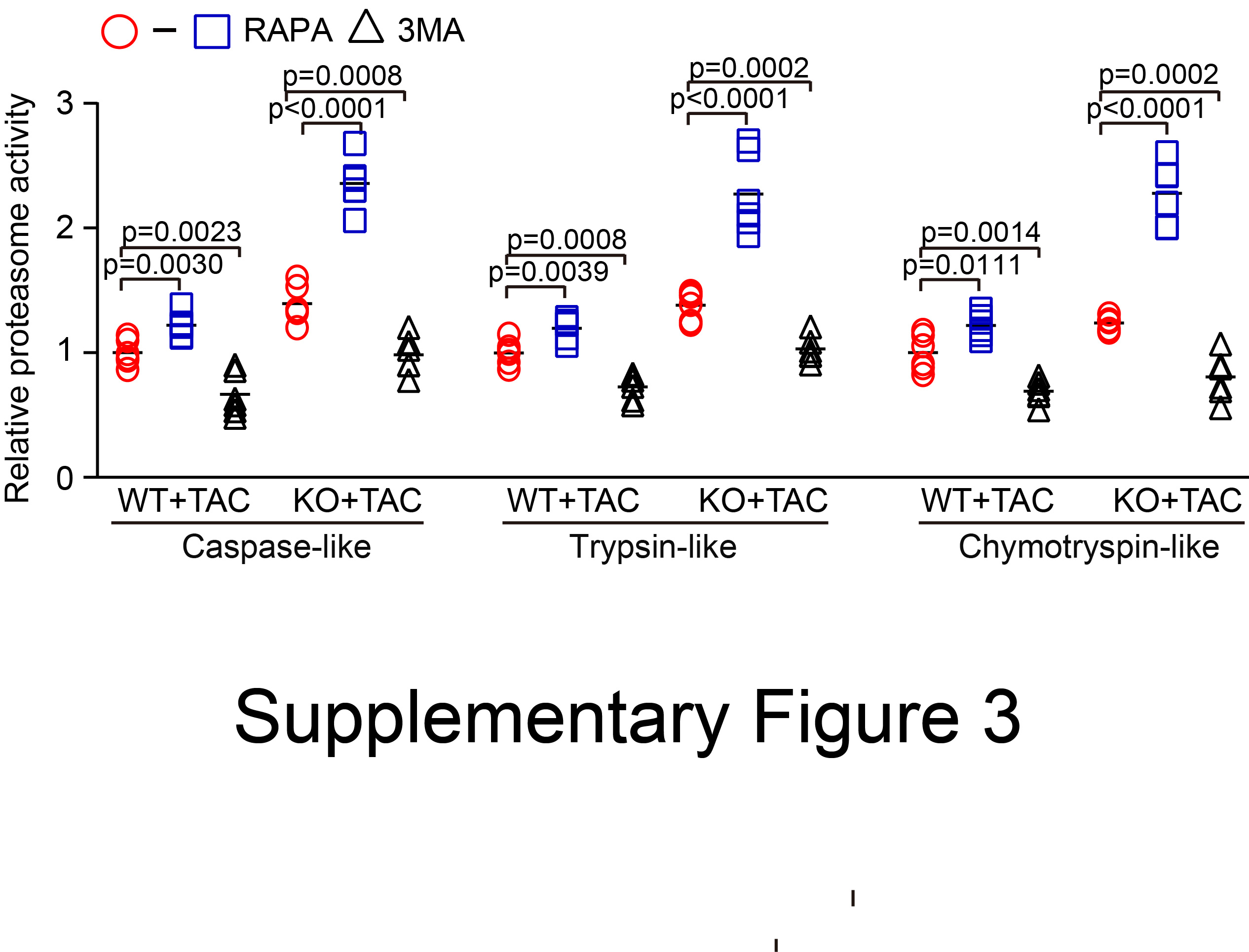

Supplement: Supplementary file 5 — Supplementary file5 (JPG 428 KB) [file 10565_2022_9699_MOESM5_ESM.jpg]
